# Human Leukocyte Antigen-Class I Alleles and the Autoreactive T Cell Response in Psoriasis Pathogenesis

**DOI:** 10.3389/fimmu.2018.00954

**Published:** 2018-04-30

**Authors:** Jörg Christoph Prinz

**Affiliations:** Department of Dermatology, University Clinics, Ludwig-Maximilian-University of Munich, Munich, Germany

**Keywords:** psoriasis, pathogenesis, autoreactive T cells, human leukocyte antigen association, HLA-C*06:02, T-cell receptor, autoimmunity, autoantigens

## Abstract

Psoriasis is a complex immune-mediated inflammatory skin disease characterized by T-cell-driven epidermal hyperplasia. It occurs on a strong genetic predisposition. The human leukocyte antigen (HLA)-class I allele HLA-C*06:02 on psoriasis susceptibility locus 1 (PSORS1 on 6p21.3) is the main psoriasis risk gene. Other HLA-class I alleles encoding HLA molecules presenting overlapping peptide repertoires show associations with psoriasis as well. Outside the major histocompatibility complex region, genome-wide association studies identified more than 60 psoriasis-associated common gene variants exerting only modest individual effects. They mainly refer to innate immune activation and the interleukin-23/T_h/c_17 pathway. Given their strong risk association, explaining the role of the HLA-risk alleles is essential for elucidating psoriasis pathogenesis. Psoriasis lesions develop upon epidermal infiltration, activation, and expansion of CD8^+^ T cells. The unbiased analysis of a paradigmatic Vα3S1/Vβ13S1-T-cell receptor from a pathogenic epidermal CD8^+^ T-cell clone of an HLA-C*06:02^+^ psoriasis patient had revealed that HLA-C*06:02 directs an autoimmune response against melanocytes through autoantigen presentation, and it identified a peptide form ADAMTS-like protein 5 as an HLA-C*06:02-presented melanocyte autoantigen. These data demonstrate that psoriasis is an autoimmune disease, where the predisposing HLA-class I alleles promote organ-specific inflammation through facilitating a T-cell response against a particular skin-specific cell population. This review discusses the role of HLA-class I alleles in the pathogenic psoriatic T-cell immune response. It concludes that as a principle of T-cell driven HLA-associated inflammatory diseases proinflammatory traits promote autoimmunity in the context of certain HLA molecules that present particular autoantigens.

## Introduction

Psoriasis is a complex T-cell mediated skin disease. Skin lesions are characterized by sharply demar-cated heavily scaling inflammatory plaques which result from T-cell driven epidermal hyperplasia. T cells infiltrating psoriasis skin lesions display a T-helper/cytotoxic cell (T_h/c_) 17 phenotype producing the T_h/c_17 signature cytokines interleukin (IL)-17A, IL-22, and IFN-γ (1, 2). They promote keratinocyte proliferation, accumulation of neutrophilic granulocytes, and the production of antimicrobial peptides and other inflammatory cytokines and chemokines. Activation and differentiation of the lesional psoriatic T_h/c_17 response is maintained by IL-23 that is produced by local dendritic cells and keratinocytes. By today, blocking IL-17A or IL-23 represents the most efficient treatment modality (3).

Psoriasis is multifactorial and involves the interaction of individual genotypes with environmental, infectious, and lifestyle factors. The human leukocyte antigen (HLA)-class I allele HLA-C*06:02 is the main psoriasis risk gene (4–6). Functional clustering of common variants associated with psoriasis highlighted the roles of interferon signaling and the NF-κB cascade and of regulatory elements related to CD4^+^ and CD8^+^ T cell maturation, development, and activation including the IL-23 pathway and T_h/c_17 differentiation (7). Thus, understanding psoriasis pathogenesis has to explain the role of HLA-C*06:02 within the complex genetic background predisposing to psoriasis. Therefore, this review will focus on the functional implications of the main HLA psoriasis risk gene, HLA-C*06:02.

## HLA-Class I Association of Psoriasis: The Main Genetic Risk

The most significant association signal observed in psoriasis genome-wide association studies that satisfied the genome-wide significance threshold of *P* < 5.0 × 10^−8^ (8) was observed within the major histocompatibility complex (MHC) region (7, 9–13). The highly polymorphic nature and density of genes and the extensive linkage disequilibrium that exists within the MHC, however, had initially hampered the identification of the causal gene that confers psoriasis susceptibility in the HLA region. Sequence-based methods in large samples finally proved HLA-C*06:02 as the main psoriasis risk gene (4, 6). HLA-C*06:02 defines early onset, severity and familial clustering of psoriasis (14–16). Unlike HLA-class II-associated autoimmune diseases psoriasis shows no evidence of interactions between different HLA alleles. HLA-C*06:02 contributes a non-additive risk effect and represents a true HLA-class I risk gene (17).

Interpreting HLA associations has to consider that the frequency of alleles can differ between populations. The prevalence and incidence of psoriasis shows ethnic and geographic variations. A relatively high prevalence of psoriasis in European countries and in the USA (0.5–6.5%) contrasts with a low prevalence in East Asian countries (0.2–0.3%) (18, 19). Given the strong risk effect of HLA-C*06:02, the ethnic allele frequency spectra of HLA-C*06:02 may at least partially explain the heterogeneity of psoriasis in different ethnic populations. This highlights the need to use population-specific reference panels made by deep sequencing to impute MHC alleles and amino acids (8). The strong psoriasis risk of HLA-C*06:02 allele has been validated in worldwide populations including Europeans (17, 20), East (6, 21), and South Asians (22), with odds ratios of as high as 3.0–10.0. HLA fine-mapping analysis using the HLA imputation method successfully identified multiple other less obviously associated HLA-class I and class II variants that confer psoriasis risk independently from HLA-C*06:02 (20). Aside from HLA-C*06:02 psoriasis associated with HLA-C*12:03, HLA-C*07:01, HLA-C*07:02, HLA-C*07:04, HLA-B*27, and HLA-B*57. Further associations were seen with *HLA-B* amino acid positions 9, 67, and 116, *HLA-A* amino acid 95, and *HLA-DQ*α*1* amino acid position 53 which are all localized within the HLA antigen binding region (6, 20, 21, 23). Although HLA-C*06:02 and the AA position 67 of *HLA*-B are shared between Caucasian and Chinese populations, other independent HLA-risk variants differ between the two populations. HLA-A*02:07, which corresponds to the cysteine residue at HLA-A position 99, shows a strong association in Chinese but is very rare or absent in Europeans, whereas HLA-B*07 shows a strong association in Caucasians while it is very rare in Chinese. The other HLA variants are common in both Caucasian and Chinese, but show population-specific associations, HLA-A*02:01 for Caucasian and the AA positions 114 and 144 of HLA-A for Chinese. These population-specific effects contribute significantly to the ethnic diversity of psoriasis prevalence (12).

The Japanese population has unique characteristics. Psoriasis prevalence in Japan is one of the lowest compared with worldwide populations (0.1–0.3%) (24–27), and HLA-C*06:02 is almost absent within the Japanese population (<0.5%) (21). Due to low allele frequency in the Japanese population, the impact of HLA-C*06:02 on psoriasis susceptibility in Japanese psoriasis patients was less apparent compared with that seen in other populations (2.3% in psoriasis cases and 0.4% in controls). Still, a Japanese-specific reference panel showed increased odds ratios for HLA-C*06:02, HLA-C*12:02, and HLA-C*07:04 in the Japanese psoriasis population (21).

Interestingly, several of the psoriasis-associated HLA-class I alleles are also significantly increased in Crohn’s disease (HLA-C*06:02, HLA-C*12:02) and ulcerative colitis (HLA-C*12:02, HLA-C*07:02) (28). HLA-B*27 predisposes for ankylosing spondylitis, inflammatory bowel disease, and psoriasis arthritis, creating an overlapping HLA-class I risk pattern although no autoantigens have been identified in these diseases.

## Functional Aspects of HLA-Class I Alleles in Psoriasis

Genetic variation in HLA genes within the MHC locus is associated with many immune-mediated inflammatory diseases (IMIDs): virtually any autoimmune condition is associated with particular HLA-class I or class II alleles (29–31). For most of these diseases, the HLA association explains more disease risk than any other gene locus. While IMIDs share many of the non-HLA loci, the associated HLA-class I and/or class II alleles are usually disease specific (32). This attributes the HLA alleles with a high degree of disease specificity and pleads for a direct causal role in the pathogenesis of the respective IMID. The association with HLA-C*06:02 in psoriasis is particularly intriguing because only 3 of more than 12,000 different HLA-class I alleles show a strong disease linkage: HLA-B*27 with ankylosing spondylitis, HLA-B*51 with Behçet’s disease, and HLA-C*06:02 with psoriasis.

Human leukocyte antigen-class I molecules are expressed by all nucleated cells. They present peptide antigens of usually 8–10 amino acids to αβ T-cell receptors (TCRs) of CD8^+^ T cells (33). The antigenic peptides are derived from cytoplasmic proteins, i.e., proteins produced within the cells. They are processed from the parent proteins by the proteasome for loading into the peptide-binding groove of the HLA-class I molecules. The complex of peptide and HLA-class I molecule is transported to the cell membrane for recognition by CD8^+^ T cells (34, 35). Accordingly, an HLA-class I-restricted immune response must be directed against a particular target cell which expresses the antigenic protein within the cytoplasm.

Human leukocyte antigen alleles are extremely polymorphic. Sequence variation is concentrated in the α1 and α2 domains that contain the binding sites for peptide antigens and interact with the TCR. HLA polymorphisms result in variable peptide-binding grooves. They contain two or three specific acceptor sites or pockets which bind specific amino acid side chains of peptides and thus define the spectrum of antigenic peptides a particular HLA molecule can present (35, 36). Due to the diverse acceptor sites, different HLA molecules select different peptide repertoires for presentation (37, 38), although the binding specificities may overlap (39, 40). Because the peptide residues in between the anchors may be flexibly occupied a single HLA-class I molecule can theoretically display between 6 × 20^5–7^ different decamer peptides (36, 41, 42).

The amino acid motifs of peptides presented by HLA-C*06:02 and several other HLA-class I molecules have recently been characterized in detail (43–45). Nonamer peptides presented by HLA-C*06:02 show select amino acids at the anchor residues 2 and 9, and a potentially secondary anchor at residue 7 (Table 1). The dominant amino acids are arginine at residue 2, leucine, valine, and less preferred isoleucine and methionine at residue 9, and arginine at residue 7. As a particular feature, HLA-C*06:02 has very negatively charged pockets defining peptides with large positively charged amino acids at residue 2 (B-pocket) and 7 (E-pocket) and thus selects a distinct repertoire of positively charged peptides. The studies of Di Marco et al. (43) and Mobbs et al. (44) further provided an estimate of the spectrum of self-peptides presented by HLA-C*06:02 and other HLA-C molecules. Depending on the experimental approach peptide elution identified between 1,000 and 3,000 different self-peptides from HLA-C*06:02 expressed in a B-cell line (43, 44). Thus, a substantial part of the cellular proteome should be immunologically visible to CD8^+^ T cells (44).

**Table 1:** Amino acids at anchor residues 2 and 9 of peptide antigens and ADAMTS-like protein 5 (ADAMTSL5) presented by psoriasis-associated human leukocyte antigen (HLA) molecules.

HLA molecule	Residue 2	Residue 9
HLA-C*06:02	R/Y/K^a^	L/V/I/Y/F/M
HLA-C*07:01	R/T/N	L/F/Y/M
HLA-C*07:02	R/Y/K	L/Y/F/M
HLA-B*27	R	L
ADAMTSL5 peptide	R	L

*^a^One letter amino acid code; font size approximately reflects the relative frequency of the respective amino acid observed at this position in peptides eluted from the HLA molecules according to Ref. (44–45)*.

According to the peptide-binding pattern HLA-C*06:02 was assigned to the same HLA supertype as other psoriasis-associated HLA alleles including HLA-C*07:01, HLA-C*07:02, and HLA-B*27 (43). All of them utilize the same anchor residues and present peptide repertoires that may partly overlap (Table 1) (43–45). HLA-C*06:02 further shares the strong negative charge of the E-pocket with HLA-C*12:03 (44) which has several similar functional domains and peptide-binding pockets as HLA-C*06:02 (46) and constitutes another potential HLA-risk allele for psoriasis and psoriatic arthritis (20, 23). Thus, several psoriasis-associated HLA-class I molecules have overlapping peptide-binding properties and might replace each other in conferring psoriasis risk. Due to the strength of association, HLA-C*06:02 may be considered the representative HLA-risk allele within this spectrum and should therefore be particularly suitable for analyzing the role of HLA in psoriasis pathogenesis.

## The Target Cell and Autoantigens of an HLA-C*06:02-Restricted Pathogenic Psoriatic T Cell Response

Heritability is an estimation of how much variation in a disease can be explained by particular genetic variants. Genetic information then has to be combined with functional analysis to allow for a precise definition of the particular role of certain genes in disease. The actual immunological role of HLA-class I molecules suggests that HLA-C*06:02 may predispose to psoriasis by presentation of an autoantigen from a skin-specific cell population. Identification of target cell and potential autoantigens of the psoriatic immune response therefore appeared as the major challenge for clarifying psoriasis pathogenesis.

This approach requires the TCRs of the pathogenic T-cell response. The paired α- and β-chains of TCRs define both HLA restriction and fine peptide specificity of a T cell (47). Because of the high diversity of the human TCR repertoire any two T cells expressing the same αβ-TCR heterodimer likely arose from a common progenitor T cell (48). In response to antigen stimulation, T cells become activated and undergo clonal expansion at the site of antigen exposure. In the obvious absence of infections, expanded TCR clonotypes are commonly viewed to characterize those T cells relevant for the pathogenesis of IMIDs (48, 49).

**Figure 1:**
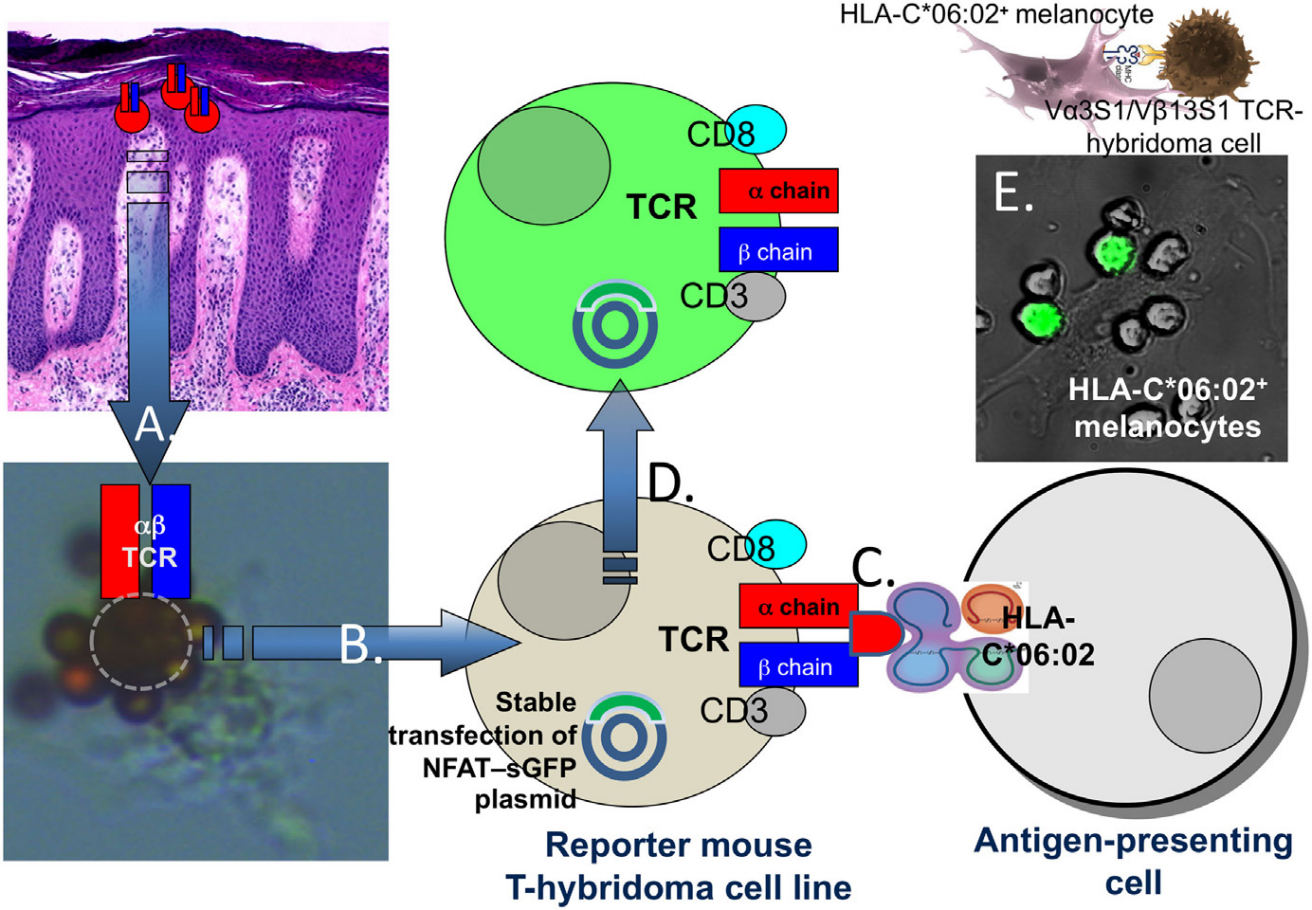
Proof of the HLA-C*06:02-restricted autoimmune response against melanocytes in psoriasis. Following separation of epidermis and dermis of lesional biopsies from HLA-C*06:02^+^ psoriasis patients CD8^+^ T cells (A) were isolated from epidermal cell suspensions using magnetic beads coated with CD8 antibodies. The arrow “A” points to a CD8^+^ T cells (encircled) isolated from lesional psoriatic epidermis which is rosetted by magnetic beads with a melanocyte attached. T-cell receptor (TCR) α and β chain mRNA of single T cells was transcribed into cDNA and sequenced by a newly developed method for single cell TCR analysis (54), cloned into expression plasmids and (B) expressed in a TCR^α−β−^ mouse reporter T-hybridoma cell line stably transfected with a plasmid for super green fluorescent protein under control of NFAT (57–59). The TCR hybridoma cells were cocultured with various primary cell types or cell lines either positive or negative for HLA-C*06:02 (C). Upon TCR ligation, the hybridoma cells produce green fluorescent protein which was visualized by UV-fluorescence microscopy or FACS analysis (D). (E) Shows the activation of the Vα3S1/Vβ13S1-TCR hybridoma by spindle-shaped HLA-C*06:02^+^ primary melanocytes in a coculture experiment.

Psoriasis lesions develop upon epidermal infiltration and activation of CD8^+^ T cells (50, 51). Marked oligoclonality of the T-cell populations within psoriatic skin lesions indicated that psoriatic T-cell activation is driven by locally presented antigens (52–56). Lesional T-cell clones were strictly associated with psoriatic skin lesions and reappeared in relapsing psoriasis (52, 56). T-cell clonality was definitely proven by single cell TCR analysis and particular evident for CD8^+^ T cells in lesional epidermis (52, 54). Accordingly, the clonal TCRs likely characterized those CD8^+^ T cells which mediate psoriatic inflammation. To identify potential targets of the pathogenic psoriatic T-cell response we expressed the paired α- and β-chains of clonal CD8^+^ T cells from the psoriatic T-cell infiltrate along with human CD8 α and β chains in a T-hybridoma cell line that reports on TCR signaling through the expression of super green fluorescence protein under control of NFAT (Figure 1) (57, 58). Consequently, these TCR hybridomas carried the specificity of the pathogenic psoriatic T cells and allowed for an unbiased, i.e., hypothesis-free analysis of the immunologic targets of the pathogenic psoriatic immune response.

Using a paradigmatic Vα3S1/Vβ13S1-TCR from an epidermal CD8^+^ T-cell clone of an HLA-C*06:02-positive psoriasis patient, we could identify melanocytes as HLA-C*06:02-restricted target cells of the psoriatic immune response (Figure 1) (59). By means of plasmid-encoded peptide libraries, we then determined the amino acid pattern of HLA-C*06:02-presented peptide ligands of the Vα3S1/Vβ13S1-TCR. Nonamer peptides that ligated the Vα3S1/Vβ13S1-TCR displayed arginine at residues 2 and 7, and leucine at residue 9 and thus corresponded to the conserved amino acid pattern which is preferentially presented by HLA-C*06:02 and other psoriasis-associated HLA-class I molecules (Table 1) (43–45). Screening the human proteome and the transcriptome of melanocytes with this particular amino acid pattern identified several peptides from natural human proteins which ligated the Vα3S1/Vβ13S1-TCR. Only a peptide from ADAMTS-like protein 5 (ADAMTSL5), however, was immunogenic in the context of the full-length parent protein and unlike the other peptides could be generated through proteasomal cleavage and NH_2_-terminal ERAP1 trimming. Knock-down and mutation experiments finally confirmed the role of ADAMTSL5 as melanocyte autoantigen (59). Blood lymphocytes of more than two-thirds of psoriasis patients but not healthy controls responded to ADAMTSL5 stimulation by production of the psoriasis key cytokines, IL-17 or IFN-γ (59). These data proved psoriasis as a true T-cell mediated autoimmune disease. They indicate that HLA-C*06:02 predisposes to psoriasis by mediating an autoimmune response against melanocytes through autoantigen presentation. The pathogenic psoriatic Vα3S1/Vβ13S1-TCR now represents a unique opportunity for understanding the immunopathogenesis not only of psoriasis but also of mechanisms of autoimmunity in general, since it is still unique in medical research: In no other autoimmune disease a similar approach has yet been successful to identify target cells and autoantigens.

The unbiased analysis of a pathogenic psoriatic TCR differs from other hypothesis-driven approaches for the identification of psoriatic autoantigens. Some of them were based on sequence homologies between proteins from keratinocytes and *S. pyogenes* (60, 61), a major infectious psoriasis trigger (62). The pleiotropic multifunctional 37 amino acid molecule LL37, which is generated by extracellular cleavage of the C-terminal part of the 170 amino acid Cathelicidin antimicrobial peptide (63) was proposed a potential autoantigen because LL37 peptides induced strong T-cell responses in psoriasis (64). Verification of the potential autoantigenic character for all these potential autoantigens was based on peptide stimulation assays. Several of the candidate peptides had been chosen according to HLA-C*06:02 anchor motifs. The insights into TCR polyspecificity, however, would predict that peptides designed this way will likely induce T-cell activation irrespective of pathogenic relevance (42). Furthermore, some of the proposed LL37 peptides did not contain the appropriate anchor amino acid residues for binding to HLA-C*06:02 (44). Without confirming that an HLA-class I-presented peptide can be generated from the parent protein by antigen processing and presentation pathways within the target cell, a role as autoantigen for CD8^+^ T cells should therefore be interpreted with care.

## Discussion

Defining the functional role of the main psoriasis risk gene, HLA-C*06:02, allowed for re-defining the architecture of the pathogenic psoriatic immune response. It proposes an HLA-centered pathogenetic model for psoriasis and other HLA-associated IMIDs where a particular HLA allele represents the causal risk gene (65). In psoriasis, HLA-C*06:02 facilitates a T-cell mediated skin-specific autoimmune response. The identification of melanocytes as organ-specific autoimmune target cells of an HLA-C*06:02-restricted immune response and of ADAMTSL5 as a melanocytic autoantigen using a pathogenic psoriatic Vα3S1/Vβ13S1-TCR provided direct experimental evidence for the autoimmune nature of psoriatic inflammation, and it explained why psoriatic inflammation primarily affects the skin (66). The predisposing HLA allele appears as an essential precondition for a tissue- and antigen-specific autoimmune response by its capacity for presentation of select autoantigens. By itself, however, autoantigen presentation is likely not sufficient for causing disease onset but requires the additive effects of common gene variants in genes which provide the costimulatory signals for activation of the actual autoimmune response. The list of common risk gene variants in psoriasis affecting proinflammatory pathways, peptide epitope trimming, IL-23 signaling and T_h/c_17 differentiation is long. It includes genes related to type I interferon signaling (*ELMO1, TYK2, SOCS1, IFIH1/MDA5, RNF114, IRF4, RIG1/DDX58, IFNLR1/IL28RA*, and *IFNGR2*), activation of NF-κB pathways (*TNFAIP3, TNIP1, TYK2, REL, NFkBIA, CARD14, CARM1, UBE2L3*, and *FBXL19*), N-terminal antigen trimming (*ERAP1*), CD8^+^ T-cell differentiation (*ETS1, RUNX3, TNFRSF9, MBD2*, and *IRF4*), and the IL-23/IL-17A axis (*IL23R, IL12B, IL12RB, IL23A, IL23R, TYK2, STAT3, STAT5A/B, SOCS1, ETS1, TRAF3IP2, KLF4*, and *IF3*). These genetic traits may augment responsiveness of innate immune mechanisms, provide a proinflammatory environment, and generate sufficient costimulatory signals which may finally exceed the thresholds for activation, differentiation and maintenance of the pathogenic autoreactive T-cell response in psoriasis (65, 66). Overall, these insights support that proinflammatory genetic traits may promote autoimmunity in the presence of the appropriate HLA molecules which present a particular autoantigen.

## Author Contributions

JP has drafted and written the manuscript.

## Conflict of Interest Statement

The author declares that the research was conducted in the absence of any commercial or financial relationships that could be construed as a potential conflict of interest.

## Abbreviations

TCR, T-cell receptor; GWAS, genome-wide association study; HLA, human leukocyte antigen; MHC, major histocompatibility complex; ADAMTSL5, ADAMTS-like protein 5.
